# Biogenic Synthesis of Zinc Nanoparticles, Their Applications, and Toxicity Prospects

**DOI:** 10.3389/fmicb.2022.824427

**Published:** 2022-06-10

**Authors:** Simran Rani, Pradeep Kumar, Priyanka Dahiya, Amita Suneja Dang, Pooja Suneja

**Affiliations:** ^1^Plant-Microbe Interaction Laboratory, Department of Microbiology, Maharshi Dayanand University, Rohtak, India; ^2^Centre for Medical Biotechnology, Maharshi Dayanand University, Rohtak, India

**Keywords:** nanofertilizers, nano-ZnO, biogenic synthesis, microbes, drought stress tolerance

## Abstract

Nanofertilizers effectively deliver the micronutrients besides reducing the phytotoxicity and environmental damage associated with chemical fertilizers. Zinc, an essential micronutrient, is significant for chloroplast development, activation of certain enzymes, and primary metabolism. Nano zinc oxide (ZnO) is the most widely used zinc nanoparticle. Concerns regarding the toxicity of conventional physical and chemical methods of synthesizing the nanoparticles have generated the need for a green approach. It involves the biogenic synthesis of metallic nanoparticles using plants and microorganisms. Microbe-mediated biogenic synthesis of metallic nanoparticles is a bottom-up approach in which the functional biomolecules of microbial supernatant reduce the metal ions into its nanoparticles. This review discusses the biological synthesis of nano-ZnO from microorganisms and related aspects such as the mechanism of synthesis, factors affecting the same, methods of application, along with their role in conferring drought stress tolerance to the plants and challenges involved in their large-scale synthesis and applications.

## Introduction

**FIGURE 1:**
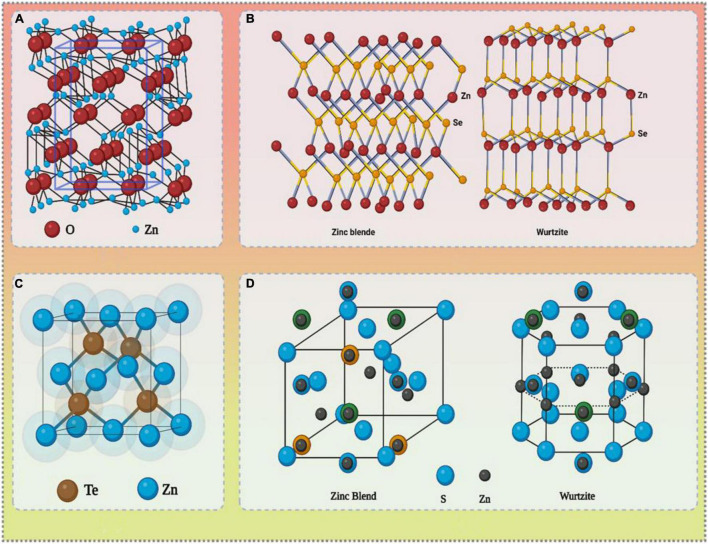
Crystal structure of various ZnNPs: **(A)** ZnO, **(B)** ZnSe, **(C)** ZnTe, and **(D)** ZnS.

Zinc is one of the most significant micronutrients required for plant growth, taken up *via* roots in the form of Zn^2+^ ions/complexes with organic acid chelates (Palmgren et al., 2008). Plants have also been found to absorb zinc forms through leaves, though the underlying mechanism has not been deciphered in detail (Doolette et al., 2018). A number of significant functions in plants are attributed to zinc, activating certain enzymes involved in the metabolism of carbohydrates, synthesis of proteins, regulation of auxin, maintaining integrity of cell membrane, and formation of pollen (Marschner et al., 1996; Umair Hassan et al., 2020). Zinc deficiency in soils and plants is a global problem because of its lower solubility in soils. Deficiency of zinc leads to stunted growth, reduced leaf size, chlorosis, spikelet sterility, and also makes vegetation more susceptible to biotic and abiotic stress, causing huge losses to the crop yield (Gondal, 2021). Some approaches to overcome Zn deficiency include using zinc-enriched chemical fertilizers, conventional breeding techniques, zinc-solubilizing bacteria, etc. But a vast majority of farmers rely entirely upon chemical supplies to augment zinc deficiency as other developments are either not popular or efficient as per the requirements of modern agriculture (Rani et al., 2020). Most of these agri-chemicals applied to the crops do not reach the target site owing to factors such as leaching, hydrolysis, microbial degradation, etc. (Ortiz-Hernández et al., 2013). Nanoparticles and nanocapsules have proved to be an effective means to distribute pesticides and fertilizers in a more controlled fashion with high site specificity (Pramanik et al., 2020). Plants are able to absorb the nanoforms of micronutrients rapidly, and also the concentration required is comparatively lesser compared to the bulk form of the same (Sabir et al., 2014). Various types of zinc nanoparticles (ZnNPs) such as zinc oxide (ZnO), zinc selenide (ZnSe), zinc ferrite (ZnFe_2_O_4_), zinc phosphide (Zn_3_P_2_), zinc telluride (ZnTe), and zinc sulfide (ZnS) find their applications in diverse arena of biomedicine, agriculture, and several other industries (Ali et al., 2018; Ansari et al., 2018; Jiang et al., 2018). Crystal structures of a few ZnNPs have been depicted in Figure 1. Significant contribution of ZnNPs has been witnessed in healthcare and agriculture owing to their antimicrobial potential, aiding targeted drug delivery, diagnostic purposes, and mitigating zinc deficiency and stress tolerance in plants (Ansari et al., 2018; Sturikova et al., 2018). Among these, nano-ZnO remains the most widely used type of NPs because of its suitable properties, easy availability, low chemical price, stability at high temperature, and neutral pH (Moezzi et al., 2012; Sturikova et al., 2018). This review comprehensively covers the microbe-mediated synthesis of zinc oxide nanoparticles (ZnONPs), intracellular and extracellular mechanisms, and various factors affecting their synthesis. Different modes of delivery with their possible role as nanofertilizers and conferring drought stress tolerance to the plants have also been discussed.

## Biogenic Synthesis of Zinc Oxide Nanoparticles

The conventional methods of synthesizing ZnONPs include physical methods such as ultrasonification, laser ablation, irradiation, and chemical methods such as microwave, pyrolysis, solvothermal, chemical reduction, and photochemical (Jameel et al., 2020). While the former requires highly sophisticated instruments besides use of highly toxic chemicals and high energy consumption, leading to the rising cost of process, drawbacks of the latter are also very well known. Non-biodegradable nature and toxicity of the chemicals restrict the application of chemically synthesized NPs, especially in the biomedical area (Patel et al., 2015). Both physical and chemical methods imply more number of steps (Salem and Fouda, 2021). These limitations have paved the way for large-scale use of biogenic synthesis that is green, safe, more economical, and requires lesser number of steps (Khandel et al., 2018).

Biogenic synthesis of metallic NPs is achieved through plants and microorganisms (Mukunthan and Balaji, 2012). The actual mechanism of synthesizing metallic nanoparticles remains the same in both. Neither it requires high-pressure/-temperature conditions nor use of toxic/hazardous chemicals. Other than being convenient, there is also no requirement of adding external reducing, stabilizing, and capping agents. Biogenic like chemical synthesis is facilitated by the bottom-up approach that involves assembling of the atoms into nuclei followed by growth into NPs. Top–down approach, on the other side, is driven by rupturing of bulk materials into fine particles as is the case with physical techniques (Salem and Fouda, 2021). In plant-mediated biogenic synthesis, the aqueous solution of metal salts consisting of metal ions is reduced by means of reducing agents that are present in the plant extract. The atoms thus obtained aggregate and form small clusters that further grow into particles (Ahmad et al., 2010; Bankar et al., 2010). Roots, shoots, flowers, seeds, fruits, bark, stems, and leaves act as different sources of biomass and secondary metabolites such as flavonoids, alkaloids, saponins, steroids, and tannins act as reducing and stabilizing agents (Abdel-Aziz et al., 2014). Plant extracts of *Lemna minor*, *Parthenium hysterophorus*, *Satureja sahendica* Bornm, and *Carissa spinarum* L. have been recently employed to synthesize ZnONPs (Del Buono et al., 2021; Umavathi et al., 2021; Chegini et al., 2022; Saka et al., 2022). Furthermore, agro wastes like coconut shell, red peanut skin extract, banana peel, and bagasse extract are being used to prepare a variety of metallic nanoparticles (Sinsinwar et al., 2018; Pan et al., 2020; Ruangtong et al., 2020; Ishak et al., 2021). However, this results in the production of polydispersed nanoparticles owing to the involvement of numerous phytochemicals that also can alter with the seasonal changes (Singh et al., 2013; Ovais et al., 2016).

**FIGURE 2:**
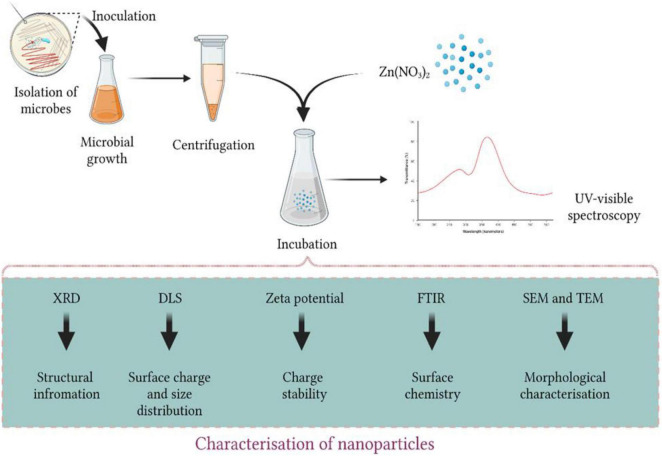
Microbe-mediated synthesis of ZnONPs.

Microbe-mediated synthesis involves reduction of metal ions into metal NPs with enzymes and other biomolecule compounds of the microbes, *viz*., bacteria, fungi, yeast, and algae (Mohd Yusof et al., 2021). Herein NPs are formed due to oxidation/reduction (O/R) of metallic ions by enzymes, proteins, and sugars (Figure 2). Each microbe interrelates with metallic ions using several pathways that, along with environmental conditions like temperature and pH, affects various characteristics of NPs such as size, shape, and morphology (Prabhu and Poulose, 2012; Makarov et al., 2014). Depending upon the type of microbe, NPs can be formed either intracellularly or extracellularly (Mohamed et al., 2019). Cofactors like NADH and NADPH-dependent enzymes, along with several compounds like naphthoquinones, anthraquinones, and hydroquinones, have been found to play a vital role in the reduction and production of metallic nanoparticles (Patra et al., 2014; Bose and Chatterjee, 2016). Intracellular mode presumes the interaction between the intracellular enzymes of microbe and the positively charged groups that leads to gripping of metal ions from the medium followed by its reduction into the cell (Dauthal and Mukhopadhyay, 2016). In microbe-mediated synthesis, it is possible to control and manipulate size and shape of NPs so as to produce the desired ones suitable for a particular application. Bacteria, entitled as the factory of NPs, are preferred for biogenic synthesis because of easy purification and requirement of mild conditions and higher yield. Filamentous bacteria, actinomycetes, have a unique advantage of secreting a wider range of secondary metabolites helping to synthesize NPs with diverse surface and size characteristics (Salem and Fouda, 2021). Attributes like highly efficient metabolites to fabricate various nanoparticles, ability to secrete well-built amounts of proteins, and easy to be traded in laboratory make fungi also to be used widely in the field of biogenic synthesis (Dhillon et al., 2012; Fouda et al., 2018; Mohamed et al., 2019). Also, fungal-mediated NPs are biologically more active compared to the other microorganisms (Yusof et al., 2019). Table 1 sums up recent studies for synthesizing of ZnONPs from different microorganisms.

**TABLE 1:** Microbe-mediated ZnONPs.

Precursor	Mode of synthesis	Microorganism	Incubation conditions	Shape	Size (nm)	References
**Bacteria**						
Zinc acetate	Extracellular	*Bacillus licheniformis* Dahb1	37°C	Hexagonal	100	Abinaya et al., 2018
		*Penicillium hibiscicola*	24 h 37°C	Spherical	60	Punjabi et al., 2018
		*Pseudochrobactrum* sp. C5	72 h 28°C	Granular	90–110	Siddique et al., 2021
	Intracellular	*Staphylococcus aureus* ATCC29213	37°C	Acicular	10–50	Rauf et al., 2017
Zinc chloride	Extracellular	*Halomonas elongata* IBRC-M10214	25–37°C	Multiform	18.11 8.93	Taran et al., 2018
		*Streptomyces* sp.	72 h 30°C	Spherical	20–50	Balraj et al., 2017
Zinc nitrate	Extracellular	*Bacillus megaterium* NCIM2326	48°h 37°C	Rod and cubic	45–95	Saravanan et al., 2018
		*Lactobacillus plantarum* TA4	24 h 37°C	Flower-like	291.1	Mohd Yusof et al., 2020
		*Sphingobacterium thalpophilum*	48 h 37°C	Rod and cubic	40	Rajabairavi et al., 2017
	Intracellular	*Lactobacillus paracasei*	Room T	Spherical	1179 137	Król et al., 2018
		*Lactobacillus plantarum* TA4	24 h 37°C	Irregular	191.8	Mohd Yusof et al., 2020
		*Pseudomonas putida* MCC2989	24 h 37°C	Hexagonal	44.5	Jayabalan et al., 2019
Zinc sulfate	Extracellular	*Alkalibacillus* sp. W7	48 h 35°C	Spherical	1–30	Al-Kordy et al., 2021
		*Bacillus cereus* MN181367	24 h 37°C	Irregular	58.77–63.3	Iqtedar et al., 2020
	Intracellular	*Lactobacillus johnsonii*	24 h 37°C	Spherical	4–9	Al-Zahrani et al., 2018
**Fungi**						
Zinc acetate	Extracellular	*Aspergillus niger*	24 h Room T	Rod and cluster	80–130	Gao et al., 2019
		*Aspergillus niger* strain (G3-1)	24 h 28 ± 2°C	Nano-rod	8–38	Mohamed et al., 2019
		*Fusarium keratoplasticum* strain (A1-3)	24 h 28 ± 2°C	Hexagonal	10–42	Mohamed et al., 2019
	Intracellular	*Aspergillus terreus* AF-1	24 h 28 ± 2°C	Spherical	10–45	Fouda et al., 2018
		*Penicillium chrysogenum* MF318506	48 h 30°C	Hexagonal	9–35	Mohamed et al., 2021
Zinc chloride	Extracellular	*Cochliobolus geniculatus*	72 h 28 ± 1°C	Hexagonal, quasi-spherical	2–6	Kadam et al., 2019
Zinc nitrate	Extracellular	*Aspergillus niger*	48 h 32°C	Spherical	61 ± 0.65	Kalpana et al., 2018
		*Aspergillus niger*	72 h Room T	Hexagonal	66	Shamim et al., 2019
		*Aspergillus niger*	10 min Room T	Spherical	76.2–183.8	Shukla et al., 2020
		*Xylaria acuta*	2 h 700°C	Hexagonal	34–55	Sumanth et al., 2020
	Intracellular	*Periconium* sp.	10 min Room T	Hexagonal	40	Ganesan et al., 2020
Zinc oxide	Intracellular	*Candida albicans*	24 h 30°C	Quasi-spherical	25	Shamsuzzaman et al., 2017
Zinc sulfate	Extracellular	*Aspergillus fumigatus* JCF	72 h 32°C	Spherical	60–80	Rajan et al., 2016
		*Aspergillus niger; Aspergillus tubulin; Aspergillus fumigatus; Penicillium citrinum; Fusarium oxysporum*	72 h 32°C	Hexagonal	30–100	Hefny et al., 2019
		*Trichoderma harzianum*	27 ± 2°C	Spherical	20–60	Kalia et al., 2020
	Intracellular	*Aspergillus terreus*	Spherical	28–63	Intracellular	Baskar et al., 2015
**Yeast**						
Zinc acetate	Extracellular	*Pichia kudriavzevii*	12, 24, 26 h 35°C	Hexagonal	10–61	Moghaddam et al., 2017
Zinc oxide	Extracellular	*Pichia fermentans* JA2	24-48h 37°C	Elongated		Chauhan et al., 2015
**Microalgae**						
Zinc acetate	Extracellular	*Chlamydomonas reinhardtii*	1 h 80°C	Nanorod, nanoflower, porous nanosheet	55–80	Rao and Gautam, 2016
		*Chlorella*	60 min 58°C	Hexagonal	19.44	Khalafi et al., 2019
		*Nostoc* sp. EA03	12 h 60°C	Stellar	50–80	Ebadi et al., 2019
Zinc nitrate	Extracellular	*Cladophora glomerata*	15 min 50°C	Spherical	14.39–37.85	Abdulwahid et al., 2019

## Mechanism of Microbe-Mediated Synthesis of Zinc Oxide Nanoparticles

**FIGURE 3:**
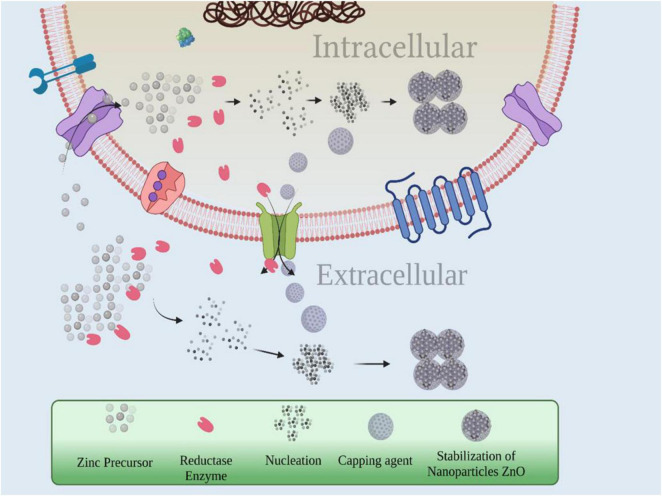
Mechanism of microbe-mediated synthesis of ZnONPs.

Microorganisms take up either of the two pathways, intracellular or extracellular, to synthesize the NPs (Figure 3). Enzymes, proteins, and other compounds produced by the microbes are used to synthesize the metallic NPs. Intracellular mechanism of synthesis involves binding of metal ions (derived from the precursor metal salt) to the carboxylate groups of specific enzymes, cysteine, polypeptides, etc., present on the cell wall of microbes; Zhang et al., 2011). It occurs as a result of electrostatic attraction between positively charged metal ions and the opposite charge carried by the carboxylate groups. Binding facilitates trapping of metal ions (Zn^2+^), which are reduced to their atomic form (Zn^0^) in the presence of bioreducing agents (enzymes) like NADH-dependent reductase. The atomic nuclei thus formed undergo growth, leading to the formation of aggregates and ultimately NPs after being stabilized by amino acids such as cysteine, tyrosine, tryptophan, and proteins/peptides (Iravani, 2014; Shedbalkar et al., 2014). NPs are found to accumulate in cytoplasm as well as cell wall (Mukherjee et al., 2001; Yusof et al., 2019).

Extracellular mechanism of synthesis is facilitated by the extracellular enzymes located on the cell membrane or released into the growth medium reducing the metal ions into atoms followed by nucleation to form NPs. Stabilization of these NPs is also facilitated by extracellular proteins secreted by the microbes that act as capping agents (Yusof et al., 2019). This enzyme has been found to be NADH-dependent reductase in *Rhodococcus pyridinivorans* TN2 by sodium dodecyl sulfate-polyacrylamide gel electrophoresis analysis. Besides reducing agents, proteins secreted by the microbes can also act as capping agents (Kundu et al., 2014).

Both these mechanisms yield white precipitates of ZnONPs, which are washed repeatedly with distilled water followed by ethanol. Drying them at 60°C for overnight yields white powder of ZnONPs. Fourier transform infrared (FTIR) spectra data indicate the bioactive molecules present on the surface of proteins such as hydroxyl, amine, and carboxyl to be the major players in reduction and stabilization of ZnONPs (Mohd Yusof et al., 2020). It has also been observed that the functional groups present in the bacterial extract act as stabilizers and capping agents in the biosynthesis of ZnONPs; therefore, there is no need to add the toxic chemicals for capping separately (Taran et al., 2017). Balraj et al. (2017) reported the synthesis of ZnONPs using *Streptomyces* sp. as a source of reducing agent. Evidence for proteins acting as reducing and capping agents was obtained from FTIR. The bands at 3,561 and 1,548 cm^–1^ can be attributed to free N–H and –C = C– vibrations assigned to amide and hydroxyl groups of amino acids and proteins, respectively, present in the cell filtrates. Extracellular mechanism of synthesis has some advantages over the intracellular one. In extracellular mechanism, NPs are synthesized in large quantities, have a comparatively simple downstream processing involving lesser number of steps, whereas intracellular one needs additional steps like harvesting CB by centrifugation and several cycles of ultrasonification for cell disruption (Markus et al., 2016).

## Factors Influencing Synthesis of Zinc Oxide Nanoparticles

A number of factors range from the type of microbial species, biosynthetic pathway, metal ion precursor, incubation time for microbial growth, and physical conditions, *viz.*, pH, temperature, reaction time. These factors lead to huge variations in the quantity and quality of NPs reflected in their characteristics such as size, shape, and stability (Patra and Baek, 2014). Distinctive characteristics are responsible for determining the unique properties of ZnONPs.

### Microbes and Their Biosynthetic Pathways

Metabolite composition of each microbe, acting as a source of reducing/stabilizing agents, is different. Also, they take up distinct pathways to synthesize the NPs of the same metal. Therefore, strain of microorganism influences the attributes of ZnONPs. Mohamed et al. (2019) reported synthesis of hexagonal and nano-rod-shaped ZnONPs from *Fusarium keratoplasticum* (A1-3) and *Aspergillus niger* (G3-1), respectively. Zeta potential is a physicochemical attribute used to determine surface charge and predict the stability of NPs (Clogston and Patri, 2011). Different species yield NPs having distinct zeta potential values. Buszewski and Pomastowski (2015) demonstrated ZnONPs yielded by *Lactobacillus plantarum* and *R. pyridinivorans* with zeta values of –15.3 and –15.5 mV, respectively. NPs having zeta potential value between +30 to –30 mV are considered unstable having higher tendency of aggregations (Kundu et al., 2014). Mohd Yusof et al. (2020) reported particle size and shape of the NPs to be dependent on the different pathways of synthesis from the same microbial strain by high-resolution transmission electron microscopy analysis. Extracellular synthesis of ZnONPs from the cell-free supernatant of *L. plantarum* TA4 resulted in flower-shaped NPs having 291.1 nm size, whereas intracellular synthesis from the cell biomass (CB) of the same microorganism produced irregular NPs having a size of 191.8 nm.

### Metal Ion Precursor

Type of metal ion precursor used, along with its concentration, influences the characteristics of ZnONPs. Different precursors are used to synthesize the ZnONPs like zinc nitrate, zinc sulfate, zinc acetate, zinc oxide, and zinc chloride. Fakhari et al. (2019) reported production of spherical-shaped ZnONPs using zinc acetate as precursor while using zinc nitrate resulted in nanoflowers of the same metal. Concentration of metal ion precursor is positively correlated to the particle growth at a faster rate but only to a certain extent. Beyond an optimum concentration, a gradual decrease in the average diameter of ZnONPs is reported with an increase in metal ion concentration (Al-Kordy et al., 2021).

### Physicochemical Conditions

Incubation conditions such as reaction time, temperature, and pH lead to variations in characteristics of ZnO-NPs. Moghaddam et al. (2017) found variations in size of NPs at different incubation times, *viz.*, 12, 24, and 36 h. Larger particle size was gained at higher incubation time. With regard to crystal shape and structure, agglomerated and low crystallinity particles with mean size of 10 ± 2.08 nm were synthesized at 12 h incubation time. Increasing the incubation time to 24 h resulted in polydispersed nanostructures with hexagonal shape and high crystallinity with average particle size of 32 ± 4.7 nm. Extending this period up to 36 h resulted in irregularly shaped and agglomerated NPs with 59 ± 10.6 nm size. Overall, 24 h incubation time was found to be most suitable by X-ray diffraction and transmission electron microscope results. Different reaction times also affected surface layer of Zn–O bonds, and hence properties of ZnONPs were altered as confirmed by FTIR data. Rao and Gautam (2016) observed the influence of temperature on the synthesis of ZnONPs using *Chlamydomonas reinhardtii* that were found to undergo a change in their shape from nanorod to nanoflowers at elevated temperature. It was because of the partial decomposition of capping agents (algal proteins) resulting in self-assemblage of nanorods into sheet-like structures and then into porous nanoflowers. A previous work by Tripathi et al. (2014) drew same conclusion during the synthesis of ZnONPs from *B. licheniformis* MTCC9555. Al-Kordy et al. (2021) optimized the synthesis of ZnONPs from *Alkalibacillus* sp. W7 and concluded that unsuitable temperature and pH leads to the deactivation of Zn^2+^ reducing enzymes, leading to larger-sized NPs. It is supported by other studies of plant-mediated synthesis of ZnONPs (Bala et al., 2015). They reported variations in size as well as morphology of ZnONPs with change in temperature from those having irregular morphology and low crystallinity at 30°C incubation transforming to dumbbell-shaped and highly crystalline at 100°C owing to increased nucleation rate of crystal formation at higher temperature. pH of the solution medium influences size as well as texture of the synthesized nanoparticles. Selvarajan and Mohanasrinivasan (2013) reported the synthesis of ZnONPs by *L. plantarum* VITES07. Membrane-bound oxido-reductases and carbon source in the culture medium acting as reducing agent have already been found to be sensitive to the variations in pH (Prasad and Jha, 2009). SEM analysis also proved that with an increase in the reaction time of ZnO nanocomposite biosynthesis the amount of particles increased and became more regular toward spherical shape.

A number of researchers have optimized the factors affecting the synthesis of ZnONPs. Ebadi et al. (2019) optimized the synthesis of ZnONPs from the cell extract of cyanobacterium *Nostoc* sp. EA03. Maximum production of ZnONPs occurred at pH 9 rather than at the lower values, and 1,000 μl cell extract concentration (1,000–3,000 μl) came out to be most suitable. Palmitic acid was suggested to play an important role as a reducing agent during synthesis. Taran et al. (2017) used Taguchi method to optimize the biosynthesis of ZnONPs by *Halomonas elongata* IBRC-M 10214 by evaluating the effects of ZnCl_2_ (zinc ion precursor) concentration, glucose concentration, and incubation temperature as controllable factors with three levels for the preparation of ZnONPs. Highest effects were observed to be due to culturing time (level 3), incubation temperature (level 3), and ZnCl_2_ concentration (level 2). A study by Fouda et al. (2018) showed the impact of precursor (zinc acetate) concentration (0.5–2.5 mM) on biosynthesis of ZnONPs. Increasing the concentration up to 2 mM indicated high efficiency of bioactive molecules on ZnONPs formation, but with a further increase they are unable to protect the formed NPs from agglomeration, leading to the aggregates of bigger size and significant decrease in absorbance. Highest productivity at maximum absorbance was observed at pH 10. This is due to more stability and reactivity in alkaline medium rather than acidic (Yedurkar et al., 2016). Incubation period varying from 1 to 5 days indicated 2 days incubation to be the best duration as highest concentration of bioactive molecules in biomass filtrate was observed. But bioactive molecules were either degraded/deactivated or affinity of fungal biomass to release these bioactive molecules reduced above 2 days of incubation age.

Besides these mentioned factors, methods of purification like centrifugation, chromatography, surrounding environment, exposure to light, and storage conditions also influence the physical structure and density of ZnONPs post-microbial synthesis (Patra and Baek, 2014).

## Mode of Delivery of Zinc Oxide Nanoparticles

**FIGURE 4:**
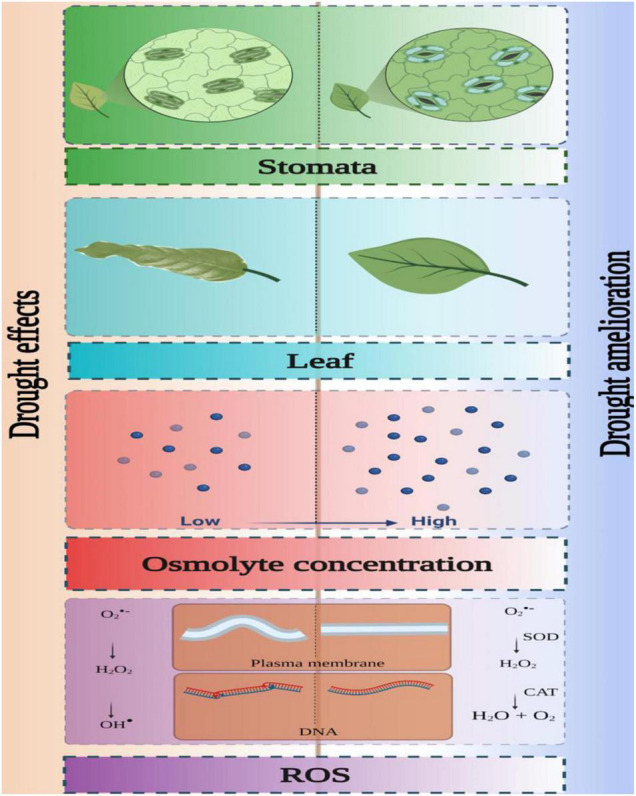
Drought stress alleviation by ZnONPs.

Disproportion between natural resource, water, and exponentially increasing human population, especially post-industrialization, has made water scarcity a global problem. Climate change-associated global warming is expected to aggravate this problem as a response of an increase in temperature. Stress led by heat and drought is quite interconnected (Zandalinas et al., 2017). Drought stress impacts plant growth and productivity, adversely taking a toll on the percentage of seed germination, transpiration rate, net photosynthetic rate, leaf relative water content, reactive oxygen species overproduction, translocation of nutrients, etc. Conventional approaches such as plant breeding, application of substances like glycine betaine and nitric oxide, using plant growth promoting endophytic bacteria, along with seed priming techniques and transgenics, have been employed to induce drought stress tolerance in plants (Ilyas et al., 2021; Rani et al., 2021). A number of studies have illustrated the role of nanoparticles in inducing drought stress tolerance in plants. Sufficient number of studies have reported the amelioration of drought stress in flora by applying ZnONPs in optimized concentration and different modes of delivery (Figures 3, 4 and Table 2). Different methods of delivering the nanoparticles have been known to result in varied outcomes. Although there is no clarity about the most suitable method of application.

**TABLE 2:** ZnONPs in drought stress mitigation.

Mode of delivery	Concentration of Nano-ZnONPs	Plant	Traits improved	References
Soil	25 mg/kg 50 mg/kg 100 mg/kg	*Triticum aestivum*	Eliminated Cd contamination alone and under water limited conditions as well	Khan et al., 2019
	1, 3, 5 mg/Kg	*Sorghum bicolor* var. 251	Fortification of many essential nutrients that were impeded under drought	Dimkpa et al., 2019
	0 mg/Kg 40 mg/Kg 80 mg/Kg 160 mg/Kg 400 mg/Kg	*Glycine max* cv. Kowsar	Concentration dependent influence on seed yield, lipid peroxidation and various antioxidant biomarkers	Yusefi-Tanha et al., 2020
	100 mg/l	*Zea mays*	Alleviated photosynthetic pigment degradation and benefited stomatal movement maintaining higher net photosynthetic rate and increased water use efficiency, enhanced starch and sucrose biosynthesis and glycolysis metabolism in leaves	Sun et al., 2021
	50 μM	*Oryza sativa* L.	Counteraction of PEG-induced drought stress	Upadhyaya et al., 2020
	0.3% w/v of distilled water (Nanochelated Zn and Fe)	*Glycine max*	Improved physiological parameters and yield	Vaghar et al., 2020
	100 mg/l	*Triticum aestivum*	Improved wheat growth and biomass, chlorophyll contents, antioxidant enzymes activity and reduced Cd uptake under Cd and drought stress	Bashir et al., 2021
	50 ppm 100 ppm	*Solanum melongena* L.	Relative water content, fruit yield and membrane stability index	Semida et al., 2021
Foliar	1.5 g/l (Zn NPs) 2 g/l (Fe NPs) Zn + Fe NPs	*Phaseolus vulgaris* L.	Traits enhanced to the maximum in case of simultaneous application	Fatollahpour Grangah et al., 2020
	50–100 ppm	*Linum usitatissimum* L.	Increment in all straw, oil and fiber traits	Rashwan et al., 2021
	1000 ppm	*Helianthus annuus* L.	Reduced symptoms of water stress severity and improvement in all growth parameters	Al-Dhalimi and Al-Ajeel, 2020
	5–10 g/l	*Vigna radiata*	Enhanced yield and yield components	Shojaei and Makarian, 2014
	50–150 mg/l (Nano-ZnO) 150–300 mg/l (Nano-Si)	*Mangifera indica* L.	Increased leaf NPK content, total carbon, sugar, proline, SOD (Superoxide dismutase), POX (Peroxidase), CAT (Catalase)	Elsheery et al., 2020
	0–100 mg/l	*Triticum aestivum*	Reduced oxidative stress and Cd contents, improved chlorophyll contents, Zn content, tolerance to both drought as well as Cd stress	Adrees et al., 2021
	0–6 mM (Zn and SA)	*Foeniculum vulgare Mill.*	Increased gram yield as well essential oil percentage	Heydarnejadiyan et al., 2021
	ZnNPs CuNPs	*Triticum aestivum*	Changes in plant morphometric indexes, leaf area, relative water content, changed ratio of chla/chlb in leaves, increase in carotenoids and SOD and CAT activity	Taran et al., 2017
Seed priming	500 ppm 1000 ppm 1500 ppm	*Triticum aestivum*	Moisture stress tolerance by maintaining membrane stability and higher expression of Cu/Zn SOD	Rameshraddy et al., 2017
	0 mg/Kg 40 mg/Kg 80 mg/Kg 160 mg/Kg 400 mg/Kg	*Glycine max* cv. Kowsar	Concentration dependent influence on seed yield, lipid peroxidation and various antioxidant biomarkers	Yusefi-Tanha et al., 2020
	50 mg/l	*Glycine max*	Helped to adapt drought stress at early vegetative stages, increased expression of tested drought tolerance marker genes	Linh et al., 2020

### Soil

Amending the soil with NPs is one of the most widely used methods for the application of NPs. Dimkpa et al. (2019) reported biofortification of many essential nutrients such as N, P, K, Zn, and Fe in *Sorghum bicolor* grown in soil amended with various concentrations of ZnO per kg of soil. This step can be considered as optimum for the production of nutrient-rich crops. Khan et al. (2019) observed nano-ZnO applied to soil, resulting in elimination of cadmium contamination along with conferring drought stress tolerance. Table 2 cites studies demonstrating alleviation of drought by amending ZnONPs in soil at various concentrations.

### Foliar

Foliar application of NPs is an advancement of foliar fertilization (Solanki et al., 2015). Foliar sprays are quite convenient for field use because they can be fed to the plants in a more controlled manner compared to the salt fertilizers, thereby reducing the toxicity symptoms *vis-à-vis* soil application (Subramanian et al., 2015; Kah et al., 2018). Foliar application of ZnONPs particularly yields more favorable results compared to conventional zinc salt formulations because of its enhanced ability to penetrate the leaves (Rossi et al., 2019). NPs with diameter of less than 100 nm can easily penetrate through the stomata of leaves and redistributed to stems through phloem sieve elements (Wang et al., 2013). Generally, it is recommended that NPs should not be used at higher concentrations owing to the toxicity aspects, but in a study by Rashwan et al. (2021), foliar sprays of ZnONPs (control, 50 ppm, 100 ppm) on flax cultivars resulted in enhancement of all straw, oil, and fiber traits such as length, yield under drought, and best results were obtained at highest concentration of ZnONPs (100 ppm). Effect of foliar application of nano- and non-nano ZnO (5 and 10 g/L), compared in *Vigna radiata*, leads to better yield components like number of pods per plant, seeds per pod, and 1,000-seed weight under water stress (Shojaei and Makarian, 2014). Besides drought stress tolerance, simultaneous alleviation of heavy metal (Cd) tolerance has also been observed by applying nano-ZnO by reducing Cd uptake and oxidative stress (Adrees et al., 2021). Combined application of nano-ZnO and other NPs like nano-Fe resulted in improved morphological and physiological traits in *Glycine max* and *Phaseolus vulgaris* (Fatollahpour Grangah et al., 2020; Vaghar et al., 2020). Salehi et al. (2021) demonstrated foliar application of ZnONPs and ZnSO_4_ to produce more remarkable effects on plant metabolism and performance compared to soil application evident from the morpho-biochemical and omics analysis of *P. vulgaris.* Foliar spray was reported to be much better than seed priming in a study by Poornima and Koti (2019). Seeds and leaves of *S. bicolor* treated with nano-ZnO and ZnSO4 showed foliar spray of ZnONPs (500 ppm) to yield highest plant height, leaf area, leaf area index, total dry matter, etc. Similar conclusion was achieved by comparing the foliar and seed application results of nano-ZnO, zinc chelate, and zinc sulfate on pinto bean (Mahdieh et al., 2018).

### Seed Priming

Treating the seeds with different organic or inorganic chemicals is referred as seep priming. It is known to cause many beneficial effects to the plants most important of which is to alleviate the adverse impact of abiotic stress on the crops (Kamithi et al., 2016). It triggers pre-germinative metabolism that helps the seeds to sustain under abiotic stresses (Paparella et al., 2015). Various techniques of seed priming involve hydropriming, biopriming, osmopriming, halopriming, hormopriming, nutripriming, solid matrix priming, redox priming, chemical priming, and nanopriming (Sher et al., 2019). Seed priming in tandem with nanoparticles treatment is called nanopriming (Mahakham et al., 2017). ZnONPs have been applied as seed priming agents in many crops till date, resulting in a significant positive influence on plant growth. One particular advantage of nanopriming is that it requires quite less amount of NPs, thus reducing the cost of production as well as environmental degradation compared to foliar and soil application. Seed priming has been to be found better than foliar application because of its enhanced ability to vigor seed germination (Khalid et al., 2021). Priming seeds of *Triticum aestivum* with 10 mg/L ZnONPs for 18 h positively influenced seed germination and seedling vigor index and enhanced seed water uptake, resulting in increased activity of α-amylase (Rai-Kalal and Jajoo, 2021). Substantial increase in antioxidant enzymes, total soluble sugar, and amylase activity noticed in rice seeds primed with ZnONPs (20–40 mg/L) with lower concentration was found to be more beneficial compared to the other treatments (Sharma et al., 2021). Nanopriming with a combination of nano-ZnO and nano-Cu leads to increase in antioxidant-scavenging machinery and yields related traits in *T. aestivum* (Taran et al., 2017). Enhanced expression of drought tolerance marker genes was observed on priming *G. max* seeds with nano-ZnO for 30 min (Linh et al., 2020).

### Nano- Versus Bulk-Nano Zinc Oxide: Efficiency and Toxicity

One of the biggest fallbacks of modern agriculture is the large-scale use of chemical fertilizers for production enhancement to meet the demand of the growing population (Chhipa, 2017). Environmental consequences, along with the loss caused by runoff/leaching/evaporation and lower macronutrient use efficiency, in case of chemical fertilizers, has shifted the focus toward nanofertilizers (Ha et al., 2019; Manjunatha et al., 2019). Nanofertilizers are well acknowledged for their target-specific and controlled release, quick diffusion, and higher nutrient use efficiency (Mikkelsen, 2018). Requirement of lesser amount of product for application than the common bulk fertilizers reduces the cost of production, phytotoxicity, and environmental damage (Davarpanah et al., 2016; Rossi et al., 2019). Nanometer size of NPs enables them to conveniently pass through cell wall and plasma membrane of plant cell and enter cytoplasm as well as organelles, thus affecting a series of metabolic processes of plants (Nair et al., 2010).

Many studies have already been taken up to analyze and compare the effects of ZnO as nano and bulk form in terms of their specificity, uptake, plant growth promotion, and stress tolerance. Bulk ZnO refers to the conventional powder comprising ZnO particles of more than 500 nm in size (Nemček et al., 2020). Adhikari et al. (2016) treated seeds of different crops, *Zea mays, G. max, Cajanus cajan, Abelmoschus esculentus*, with nano as well as micron scale of ZnO powder. The uptake of zinc was found to be more from nanoscale ZnO. Zinc-deficient agro-economies could benefit with this concept and get better produce in return. ZnONPs were found to influence some bacterial lineages in rhizospheric bacterial community to a greater extent than bulk form, which explains the varying growth response of *Lactuca sativa* L. to different forms of zinc (Xu et al., 2018). Mazaheri Tirani et al. (2019) treated hydroponically grown tobacco plants with nano and bulk zinc oxide and observed that nano-ZnO at all levels (0.2–25 μM) positively influenced the growth and anatomical attributes, whereas bulk ZnO produced such results only at a certain concentration (1 μM). Khalid et al. (2021) compared the effects of conventional and nano-enabled zinc fertilizers on morphological and physiological attributes of *Caesalpinia bonducella* wherein nano-zinc fertilizer led to higher increase in morphological parameters and chlorophyll content than the bulk form. Dimkpa et al. (2020) reported requirement of lesser Zn application rate in case of nano-ZnO to achieve food fortification with Zn. A similar degree of increase in shoot’s Zn uptake and grains’ Zn concentration was observed with nano-ZnO and bulk ZnO. Nano-ZnO increased the contents of glycyrrhizin, total phenolic compounds, and anthocyanins in licorice (*Glycyrrhiza glabra* L.) seedlings compared to bulk form. These secondary metabolites are involved in the defense mechanism of plants under stress (Oloumi et al., 2015). Only nano-ZnO-treated sample was found to enhance content of chlorophyll and protein in *T. aestivum* Linn. (Ramesh et al., 2014). Alabdallah and Alzahrani (2020) found foliar application of both bulk and nano-ZnO form alleviating the impact of salinity on okra as a result of increased stress tolerance conferring attributes such as photosynthetic pigments, SOD, and CAT activity, lowered proline, and total soluble carbohydrate concentration but ZnONPs showed more effective results. Sheoran et al. (2021) reported least leaching in case of nano-ZnO, which was utilized by the plants in maximum exchangeable form for soil and leaves compared to bulk/chemical zinc acetate.

Although these studies support the very efficiency of nano-ZnO over bulk form, but the toxicity studies of the same is also not to be ignored. Mansoor et al. (2019) demonstrated ZnONPs to have better stimulatory effects on *T. aestivum* than bulk and control but only at lower concentrations. It supports a previous study by Ramesh et al. (2014) in which higher concentration of ZnONPs impaired the seed germination in the same crop. Singh et al. (2018) demonstrated that the treatment of rice seedlings with both nano- and bulk-ZnO. Application of nano-ZnO resulted in higher biomass, higher values of PS-II kinetics, and lower values of energy fluxes parameters. But a concentration above 50 ppm caused unfavorable effects on the growth of the same pointing toward the phytotoxicity caused by ZnONPs. A number of studies have revealed that the level of toxicity by both nano-ZnO and bulk-ZnO does not have significant variation. There is an obvious need to undertake targeted studies so as to get clear statistics of toxicity.

## Limitations and Future Prospects

The major limitation rendering microbe-mediated green synthesis is that the microorganisms might lose their ability to synthesize NPs owing to the mutations over a time period (Narayanan and Sakthivel, 2010). Also, all the microbes are not able to synthesize ZnONPs; therefore, the ones having potential needs to be explored taking up rigorous screening programs (Yusof et al., 2019). The exact mechanism of microbe-mediated synthesis of NPs by both extracellular and intracellular mechanisms is yet to be unraveled. Current information available about the reducing agents and capping agents involved in bioreduction and stabilization, respectively, is still scarce. Their identification and role in determining shape and size of NPs is not clear (Rai et al., 2011). Microbe-derived NPs are not applicable for large-scale production due to the requirement of completely aseptic environment and special maintenance (Dhuper et al., 2012). Scaling up this production needs detailed optimization studies so as to unravel the influence of each and every factor. To commercially harness microbe-mediated ZnONPs, the coordination between major stakeholders that are basic sciences, chemical engineering, and industries is necessary (Salem and Fouda, 2021).

Complete biomass filtrate having metabolites involved in the synthesis of ZnONPs must be analyzed to unfurl the biochemical and molecular aspects associated with microbe-mediated synthesis. A constant quest for exploring and screening novel microbes for ZnONPs synthesis is required (Yusof et al., 2019). There is a need for more critical evaluation of nanoagrichemicals against their conventional analogs to assess the benefits and risks associated with their use (Kah et al., 2018). More studies targeted specifically on the toxicity aspects of ZnONPs should also be taken up to facilitate appropriate use of ZnONPs and encourage its large-scale cautious application.

## Author Contributions

PS and AD conceptualized the theme of this review. SR wrote and compiled the original draft. PK and PD drafted the figures and compiled tables. All authors have made intellectual and substantial contribution and approved it for publication.

## Conflict of Interest

The authors declare that the research was conducted in the absence of any commercial or financial relationships that could be construed as a potential conflict of interest.

## Publisher’s Note

All claims expressed in this article are solely those of the authors and do not necessarily represent those of their affiliated organizations, or those of the publisher, the editors and the reviewers. Any product that may be evaluated in this article, or claim that may be made by its manufacturer, is not guaranteed or endorsed by the publisher.
